# The Brazilian Green Revolution

**DOI:** 10.1016/j.polgeo.2021.102574

**Published:** 2022-01-10

**Authors:** Ryan Nehring

**Affiliations:** Department of History and Philosophy of Science, University of Cambridge Free School Lane, Cambridge, Cambridgeshire, United Kingdom CB2 3RH

## Abstract

This article analyzes the critical geopolitics of knowledge production in Brazil during the 20^th^ century. It offers a critical appraisal of recent calls to decolonize political geography by locating the role played by actors and institutions in the Global South within the broader narrative of the Green Revolution. Historical accounts of the Green Revolution have only recently started to incorporate perspectives of and attribute agency to actors in the Global South. However, Brazil has largely been left out of the geographical scope of the Green Revolution. This article focuses on U.S.-Brazilian geopolitical relations behind an effort to reproduce the U.S. model of higher education, rural extension and agricultural research in Brazil. I argue that the confluence of Brazil’s geopolitical importance with opportunities for foreign investment in its agricultural sector brought together U.S. and Brazilian experts and expertise to modernize Brazilian agriculture. The case of transnational soybean research illustrates the importance of this relationship in transforming Brazil’s agricultural sector and limiting alternatives. This article offers an account of how the geopolitics of knowledge production shape the long-term institutional legacies of national research institutions.

## Introduction

In 2006, the World Food Prize was granted to two Brazilians, Alysson Paolinelli and Edson Lobato, and to an American soil scientist named Andrew Colin McClung. They won the award for their work on “fixing” the acidic soils of the Brazilian savannah, a region known as the Cerrado. Their research made large-scale grain production both possible and profitable in tropical savannahs (see Márcio da Silva, 2012). Norman Borlaug, known as the “father of the Green Revolution” had called the Cerrado “the world’s most important agricultural expansion zone of the [20^th^] century”. At the award ceremony, the then-president of the Brazilian Agricultural Research Corporation (Embrapa), Dr. Silvio Crestana, gave a speech to describe how international collaboration between the awardees was an integral to rewriting historical national divisions of technological development. “We have a new map of the world today,” he said; “…it’s not just geographical, the nation’s borders, but [based on closing] the technological gap…; eventually, the Cerrado technology, or one similar to it, will move into the *llanos* in Colombia and Venezuela and hopefully – hopefully – into central and southern Africa^1^”.

Crestana’s remarks drew on a popular narrative in which national public research (through Embrapa) overcame structural barriers in technological development to become considered a world leader in agricultural innovation (see Klein and Luna, 2018). In fact, Embrapa, the country’s public agricultural research agency, labels itself as the “world’s leader in tropical agricultural technology”. His invocation of a “borderless” map is particularly telling because, although the story involves national pride, it is also a story of how the poles of those “with knowledge” and those “without knowledge” are continually shifting within transnational networks.

This article builds on the critical geopolitics of knowledge production. I follow Gustavo Oliveira (2018) by understanding critical geopolitics as those that, “situate power not in the hands of sovereign states alone, but in more relational ways that traverse a spectrum of scales and struggles” (350). The case of the Brazilian Green Revolution contributes to critical geopolitics by explaining the ways in which national political contexts shape questions of ownership over transnational flows of knowledge and technology. I call attention to the importance of geopolitical relationships of power in the reproduction of what Radcliffe and Radhuber (2020) call “colonial-modern dynamics of knowledge production” (3). However, rather than relying primarily on national or regional contexts, this article analyzes the institutional and transnational networks that shape the role of science and technology in agricultural development. That is not to say that national contexts do not matter. Decades of research on the postcolonial condition has attempted to privilege non-Western perspectives on social and cultural forms of development. Naylor et al. (2018) claim that postcolonial approaches still rely on a “western/non-western binary that privileges the west” (200). By focusing on the international interests and transnational networks of knowledge production, I highlight the geopolitical and institutional legacies that transcend western/non-western binaries.

The case of Brazil poses a challenge for postcolonial perspectives (see Gomes, 2011). As the largest country in Latin America, and the only former Portuguese colony in the region, Brazil experienced what Néstor Canclini (2000) describes as “exuberant modernism and deficient modernization” (41) throughout the 20^th^ century. The Brazilian state has long been both a supporter and suppressor of international knowledge exchanges, depending on their service to state objectives. As Ferretti (2018) has shown, geographers critical of the Brazilian military government (1964-1985) were exiled due to their political beliefs. During exile, these geographers were able to build and to utilize international scholarly networks in order to avoid being marginalized at home, while also contributing postcolonial perspectives abroad.

Professional bureaucrats in Brazil, on the other hand, were supported with public funds to build state bureaucracies that supported the development of the country’s interior. Wendy Wolford (2016) has shown how the National Institute of Colonization and Agrarian Reform (INCRA) developed institutionally out of the Brazilian government’s aim to move “men without land to land without men [*sic*]”. While INCRA worked to move people, public research, universities and rural extension developed technologies for farmers to adapt in the transformation of previously underdeveloped areas into agro-commodity zones. In other words, the formation of the Brazilian state in the 20^th^ century was intimately linked to the expansion and modernization of agriculture. Perhaps few agricultural crops were as important as soybeans.

Brazil recently eclipsed the United States as the world’s largest producer and exporter of soybeans. The growth of soybean production in Brazil was made possible, in part, due to the technologies developed by 2006 World Food Prize winners mentioned above. Their work in ‘fixing’ the acidic soils of the Brazilian Cerrado rendered a previously underdeveloped region of the country into one of the world’s largest grain breadbaskets (see Nehring, 2016; Dutra e Silva, 2017). For the Brazilian state, soybean production has underwritten the internationalization of Brazilian agribusiness and the consolidation of commercial ties with Asian markets (Oliveira, 2018). Soybean production in the country is now so lucrative that U.S. farmers have started migrating to the Brazilian Cerrado in search of new fertile frontiers (Ofstehage, 2016). I argue that the confluence of Brazil’s geopolitical importance with opportunities for foreign investment in its agricultural sector brought together U.S. and Brazilian experts and expertise to modernize Brazilian agriculture. Further, I suggest that the central role of the Brazilian state and its reluctance to combat existing structural inequalities in rural Brazil limited alternatives to agricultural modernization.

In what follows, I first provide a brief review of academic literature on the Green Revolution and a rationale for what Brazil can contribute to the broader Green Revolution narrative. I then turn to an overview of Brazilian-U.S. relations in relation to agricultural development in the 20^th^ century Brazil. The next section draws on the case of transnational soybean research as perhaps the most geopolitically consequential crop based on transnational agricultural science in Brazil. The Brazilian model of agricultural research gained fame due to its supposed scientific ability to create a globally competitive industrial agriculture sector in the tropics. This article draws on archival research conducted at the Central Archive of the Ministry of Agriculture, Livestock and Supply and Embrapa’s Institutional Archives, both in Brasilia. The article is also part of a broader research project based on 18 months of ethnographic research at 11 of Embrapa’s research centers throughout Brazil and five oral histories with U.S. agricultural scientists who worked in Brazil during the middle of the 20^th^ Century.

## Green Revolutions: Geopolitics and the (RE)Circulation of Knowledge

Academic literature on the Green Revolution covers extensive ground on some of the most notable cases from Latin America to South and Southeast Asia (c.f. Fitzgerald, 1986; Feder, 1976; Niazi, 2009; Hossain, 1988). There has also been significant praise of productivity increases claimed as a result of the Green Revolution. (Conway, 1997; Davies, 2003; Evenson and Gollin, 2003). Critiques of the Green Revolution have ranged from the imposition of markets (Patel, 2013) to the reproduction of social and economic inequalities (Cleaver Jr., 1972; Pearse, 1980), effects on hunger (Cullather, 2010), its role in national development (Griffin, 1974; Dahlberg, 1979) and its environmental consequences (Farmer, 1979; Shiva, 2016). But most scholars agree on the basic tenets of the Green Revolution: to use Western science as a so-called “objective” and “rational” organizational principle to maximize efficiency of “Third World” agriculture.

The Green Revolution had several objectives. Originally understood to be a “war on hunger”, further critical research has challenged any such underlying humanitarian goals. Perkins (1997) and Cullather (2010) have carefully analyzed how the Green Revolution was part of a broader geopolitical strategy of the U.S. government to subvert rural unrest and revolutionary tendencies in the developing world. U.S. government agencies worked with philanthropic organizations to target countries where there were willing participants: farmers who lived in well-irrigated areas and were receptive to adopting high-yielding commercial crops. When nation-states are included within the narrative of the Green Revolution it signifies being a consensual or coerced participant in U.S.-led development projects. But, perhaps more importantly, it also opens up the possibility to identify and contest foreign logics of Western agricultural science.

According to Harwood (2019), when scientists managed to increase farmer uptake of high-yield crops, it served to “commercialize” the countryside such that production shifted from subsistence production to “markets, sales and profits” (see also Cullather, 2006; Sackley, 2011). Similarly, Nally and Taylor (2015) have shown how these programs were based on the assumption of “self-help”, where technical matters such as increasing productivity would only succeed “if they also instilled modern mind-sets and habits in individual farmers” (61). Green Revolution programs outlined a crucial role for U.S. scientists as protagonists. For example, George Harrar grew up in New York and held degrees from Oberlin College, Iowa State and a PhD in plant sciences from the University of Minnesota. He was asked by the Rockefeller Foundation to serve as the first director of the Mexico Agricultural Program (MAP), considered to be the “Birthplace of the Green Revolution” (Harwood, 2009; 2020). Harrar then went on to the Philippines where he helped to establish the International Rice Research Institute (Chandler Jr., 1992). And perhaps the most well-known of them all, the Nobel Peace Prize laureate Norman Borlaug, “is synonymous with the Green Revolution” (USAID, 1982: 3). Actors in the Global South are too often framed as passive recipients of Green Revolution technology, whether policymakers and scientists or the farmers themselves.

Historical accounts of the Green Revolution have only recently started to incorporate perspectives of and to attribute agency to actors in the Global South. Part of the reason for their absence in the broader narrative of the Green Revolution is methodological. According to Lorek (2020), Green Revolution literature has, “until recently, followed the most accessible document trail to large institutional archives… attributing too much innovation to North Americans” (94). For Gabriela Soto Laveaga (2018), scholars should look for new narratives that “examine how and when distinct microhistories among nontraditional protagonists intersect” (23). Prakash Kumar (2017) contends that even with a fixation on U.S. actors, academic literature on the Green Revolution has expanded its geographical coverage. This expansion has “globalized” the narrative, but largely ignored who was enrolled in new geographies, why, and under what political, institutional and scientific arrangements.

The revolving door of U.S.-based philanthropic and governmental organizations mobilized financial support and built strategic relationships with foreign institutions to structure the movement of scientists and biological material (Kumbamu, 2020). According to Raj Patel (2013) the Green Revolution “so clearly relied on institutions to corral and maintain a cocktail of coercion and consent within the dominant hegemonic bloc” (3). The most high-profile institutions were the Rockefeller and Ford Foundations, who worked in concert with governments to design and implement Green Revolution programs. They are often a starting point for histories on the Green Revolution to trace the political relations and interests in the most well-documented geographies (c.f. Kumar et al., 2017; Marchesi, 2017; Chandler Jr., 1992). Historical analysis on participating non-U.S. institutions has only recently more closely examined the ways in which transnational networks, national research institutions and biological material were co-constitutive of a technopolitical hegemonic bloc. Expanding a relational understanding beyond the interests and activities of the so-called “architects” incorporates the ways in which so-called “recipient” individuals and institutions also played a crucial role in shaping the process on the ground.

In comparison to other well-documented cases of the Green Revolution, the Rockefeller and Ford Foundations played a rather limited role in Brazil. The principal foreign actor in Brazil was Nelson Rockefeller. His interrelated roles as businessman, philanthropist and U.S. government official were geared to use markets and foreign capital as the vehicle for agricultural development in Brazil. Rockefeller worked closely with state and national governments in Brazil to share ownership over and the funding of agricultural development projects. This approach differed from the more top-down approach adopted by the Rockefeller and Ford Foundations elsewhere. A consolidated vision for agricultural modernization was also shared by the Brazilian state following regime change in 1964 (see Graziano da Silva, 1981; Gonçalves Neto, 1997). It is the alignment of these interests and shared agency between the actors that characterized the Brazilian Green Revolution. Green Revolutionaries in Brazil were not only U.S. nationals, they were also Brazilian.

While Brazil’s Green Revolution might be considered one of the most effective in terms of productivity growth, it is also certainly one of the most unequal. During the Brazilian military dictatorship, small farmers and peasants were dispossessed from their land by both technologies that disproportionately benefitted large landowners and direct state violence (see Bin, 2021). This is in stark contrast to other well-known cases such as Mexico (Olsson, 2017), India (Sethi, 2006), and the Philippines (Borras Jr., 2006), where land tenure was more equitable prior to foreign technical assistance. Nevertheless, Brazilian farmers who did benefit contributed to what is now widely considered to be a productivity revolution was later exported by the Brazilian government via technological packages throughout Latin America and Africa under the banner of “South-South Cooperation” (Reifschneider et al., 2016). In this way, Brazilian agricult ural science is not merely a passive recipient of “Western” science but an active agent in the reproduction of Green Revolutions worldwide^2^. Broadening the narrative of the Green Revolution to include Brazil demonstrates how geopolitics of knowledge production can shape and reshape national institutions over time. Further, by deepening the narrative to include more agency from actors in the Global South shows how non-Western scientists were, and continue to be, building their own Green Revolutions.

## Building the Brazilian Green Revolution

One of the most important post-WWII alliances between the U.S. and Brazil was the “Abbink Mission,” or the Joint Brazil-United States Technical Commission, which was established in 1948. Following the agreement to fund the construction of a steel mill in Volta Redonda, this commission provided another channel to finance the industrialization of Brazil’s economy (see Abbink, 1984). The Mission was named for the U.S. chairman, John Abbink, who stated in an interview with the *New York Times* that the “development and modernization of agriculture would go a long way toward making that nation [Brazil] prosperous” (New York Times, 1949). In the agricultural sector, Castro (1984) has shown how the development of education, research, and extension was an explicit goal of the Commission. The members sought to identify the key shortcomings in agricultural modernization and stated, “the future of Brazil’s agriculture probably depends more on a sound and adequate research program than any other one thing” (U.S. Department of State, 1949).

The Abbink Mission paved the way for a more comprehensive cooperation agreement that would finance and strategize Brazilian agricultural development. Importantly, both the Brazilian and U.S. governments sought to create linkages between industry and agriculture to spur national economy development. The U.S.-Brazil Joint Commission for Economic Development was signed on December 19^th^ 1950 and assured US$1 billion in noninflationary internal and external financing (U.S. Department of State, 1953). This new Commission worked to outline which commodity crops would be research priorities in order to increase state revenues and to feed an increasingly urban workforce. Rice, beans, corn, soybeans, and cattle were identified as the most suitable crops for such goals (USAID, 1978).

Private philanthropy also saw opportunities to expand investment opportunities for foreigners in Brazilian agriculture. Nelson Rockefeller’s personal philanthropy organization – the American International Association (AIA) – had invested over US$1 million in extension services in the country and USAID some US$300,000 over the period of 1949-64 (Barber, 1965: 128-129). This funding financed the importation of farm implements and the training for over 209 Brazilian extension workers in the U.S. from 1951-64. Rockefeller also established a for-profit company in Brazil, the International Basic Economy Corporation (IBEC) to “develop various parts of the world, to increase the production and availability of goods, things, and services useful to the lives or livelihood of their peoples, and thus to better their standards of living” (Dalrymple, 1968: 12). Nelcon Rockefeller’s AIA and his for-profit IBEC worked closely with the Rockefeller Brothers Fund, USAID and the Brazilian government to export U.S.-inspired rural development policies and to build new agro-input markets.

Official development assistance from the U.S. to Brazil in the agricultural sector was outlined in an institutional agreement between the two countries on June 26^th^, 1953. The agreement established the Technical Office of Brazilian-American Agriculture, or ETA (*Escritório Técnico de Agricultura Brasileira-EUA*), “whereby both nations could participate jointly in all phases of the planning and administration of a cooperative agricultural program” (Barber, 1965: 1). Over the 11 years of its existence, the ETA sent 33 U.S. agronomists to train 878 extension agents in Brazil (Barber, 1965). Rockefeller’s organizations and his in-country agents were involved in almost every project in Brazil – from the activities of AIA and IBEC to the management of bilateral programs. All of their work in Brazil centered on modernizing agriculture by reproducing the triad of higher education, rural extension, and agricultural research. This was based on the U.S.’s experiences of the Morrill Act (land grant universities – 1862 and 1890), the Smith-Lever Act (rural extension – 1914) and the Hatch Act (agricultural research – 1887). In the case of Brazil, each of the components within the triad would ultimately depend on national political priorities for agricultural modernization.

Brazilian scientists at public research institutions experienced first-hand the triad of education, extension and research as they embarked on Rockefeller-funded international visits. Alfonso Wisniewski, the former director of the Agricultural Research and Experimentation Institute of Northern Brazil, departed from Belém at the mouth of the Amazon for an international tour in 1969. His trip consisted of visiting Nelson Rockefeller’s IRI headquarters in New York, USAID and the USDA in Washington DC, Iowa State University, the Universities of California at Davis and Los Angeles, the University of Arizona, Texas A&M and, lastly, the heart of the Green Revolution activity in Chapingo, Mexico. In his travel report he stated that, “economic growth in American agriculture is due undeniably to the triad of education – extension – research” (Embrapa, 1969). The time he spent in the Central Valley of California was particularly impactful for Wisniewski. He remarked how the “victory of applied science and technology was applied by a class of well-favored farmers who asserted themselves into the vastness of the plains,” which was “hardly just a mere demographic expression.” As Wisniewski and his contemporaries saw it, Brazilian land and its farmers were commensurate with that of any other in the world; but without the proper political and institutional infrastructure to generate and apply modern science, the country’s agricultural sector would forever stagnate.

### Higher Education

The training of Brazilian agricultural extension workers and scientists was a central goal in U.S.-Brazilian relations. Both the U.S. Agency for International Development (USAID) and Rockefeller’s AIA were involved in this process. AID provided over $8 million from 1951-1966 to reproduce the land-grant model of using higher education for applied agricultural research and extension (AID, 1968). The inspiration for this project stemmed from agronomist Peter Henry Rolf’s visit to Brazil in 1921. He helped establish what would become one of the country’s largest and most prestigious agricultural universities, the Federal University of Viçosa (UF-Viçosa) in the state of Minas Gerais. The university appointed Rolf as the first director, and he molded UF-Viçosa around the triad of education – extension – research based on his experience of the land-grant universities in the U.S (Fernandez, 1991).

On June 29^th^, 1951, Purdue University, a land-grant institution, established a partnership with UF-Viçosa. This was an initial attempt to internationalize the land-grant philosophy of applied research in Brazil. The Purdue-Viçosa partnership served as a model for expanding additional U.S.-Brazilian academic linkages to bolster agronomic science in the country. The public land-grant universities of Wisconsin, Ohio State, and Arizona were paired up with Brazilian counterparts^3^ based on supposed ecological and climatic similarities. The project’s goal was to increase graduate-level training tenfold and to double undergraduate enrollment in the agricultural sciences from 1963-1970 (AID, 1968). For mentorship, faculty from U.S. universities were sent to the Brazilian partner universities as educational consultants and to teach courses. Funds also were spent on laboratory equipment and related research facilities. A home economics department was also established at UF-Viçosa to train women as home economists in the countryside.

Brazilian universities failed to replicate the land-grant model of applied research as that task was to be left to rural extension and agricultural research (Sanders et al., 1989). Nevertheless, the projects were successful in developing agronomic science departments across the country. UF-Viçosa remains the most well-known and prestigious agricultural university in Brazil. It became the epicenter of training rural extensionists and agronomists in the 1980s and remains so to this day. The academic cultures in the U.S. and Brazil became closely linked in a few select institutions, especially in the case of Purdue and UF-Viçosa. However, a lack of funding and uneven institutional capacity throughout Brazil left universities unable and unwilling to take on the role of applied agricultural research as had been done in land-grant colleges in the U.S. Rather, the development of agronomic science departments in Brazilian universities eventually became a stepping stone towards a career in extension or research.

## Rural Extension

Close collaboration in establishing UF-Viçosa helped to link higher education with rural extension. UF-Viçosa was where the first effort to expand extension agencies statewide occurred in 1929 (Peixoto, 2008). It was only later, from the 1940s until the late 1970s, that U.S.-Brazilian development cooperation expanded rural extension networks (see Mendonça, 2010). Nelson Rockefeller and the state of Minas Gerais were again at the heart of the early interest. It was in Minas Gerais where Rockefeller found a state government that was already convinced that the U.S. model of rural extension would make modernization possible (see Márcio da Silva, 2015). AIA served as the primary manager of rural credit and extension in the state through an agreement in 1949 with the Rural Credit and Assistance Association (ACAR – *Associação de Crédito e Assistência Rural*) (Ribeiro, 2000). Robert W. Hudgens, a longtime public servant from the U.S. Farm Security Administration was appointed director of AIA by Rockefeller. According to AIA biographer Martha Dalrymple (1968), “Hudgens argued that if supervised rural credit had worked in the U.S., why not give it a whirl in Brazil?” (40). Working with ACAR, AIA helped to push the production of particular commodities marketed by the for-profit IBEC. Supervised extension services offered credit that was often then used to purchase hybrid seeds, pig breeds, fertilizers, farm implements and other inputs sold by complementary agribusinesses in which IBEC had an economic interest (Broehl, 1968). IBEC became a majority stakeholder in a hybrid seed company, Agroceres S.A., which was the largest in the country up until the 1980s (Stal, 1993).

Although AIA was established as a non-profit, it worked hand in hand with IBEC as a for-profit modernization machine in the Brazilian countryside. As explained by Nelson Rockefeller biographers Colby and Dennett (1995):

“It seemed like old times. Nelson’s AIA was replicating in South America what his father’s General Education Board and the Rockefeller Sanitary Board had done in the American South and Midwest: promoting fertilizer, crop rotation, irrigation, sanitation and mechanized agriculture, all the ingredients of a social formula that brought agribusiness to the United States at the expense of small farming” (216).

Part of the “social formula” also included the promotion of a gendered division of labor in rural Minas Gerais (see Pinheiro, 2017). AIA contracted women from U.S. land grant colleges to serve as “home supervisors.” Their role was to organize demonstrations of household nutrition, sanitation, and domestic administration. The women often traveled with male agronomists in order to provide a full farm and household package that would “bring rural families the modern conquests of science and technics, of research and experimentation in the field of agriculture and domestic economy” (Moura, 2017: 8). Even youth were involved with the exportation of the 4-H model to Brazil by enlisting the support of 43 Peace Corps volunteers. 4-H^4^ in Brazil was translated as 4-S for *Saber, Sentir, Servir* e *Saúde,* or Knowing, Feeling, Serving, and Health.

To promote the growth of 4-S clubs, state extension agencies provided manuals on the general principles of the organization to rural families. In the manuals, “Agricultural Projects” for prospective 4-S youth outlined entrepreneurial guidelines for selecting crops to experiment on their farms. Questions such as “is there an accessible market for the project product?” and “is the area large enough for the chosen project?” helped to narrow potential crops. But the manual also made a clear recommendation: “for basic projects we suggest: hybrid corn and soybeans” (ASCAR, 1965). Funding for 4-S was initially from USAID and the Rockefeller Foundation (Márcio da Silva, 2002). However, once a national 4-S committee was founded in 1964, the private sector largely funded the initiative. Some of the largest funders included: Nestlé and Fleischmann-Royal from the food industry, Agroceres S.A. and Union Carbine from the agricultural inputs sector, Massey Ferguson farm implements and Brazilian state rural credit banks (Gomes, 2019). According to Marin (2017), “by showing themselves to be ‘friends’ of the rural youth clubs, the companies intended to build future producers and consumers of goods with a view to integrate them into the agro-industrial supply chains that were being installed in Brazil” (27). Rockefeller’s AIA was the main funder of 4-S clubs in the state of Minas Gerais via ACAR.

The international partnership between AIA and the state government of Minas Gerais gained traction country-wide. It helped that the then-governor of the state, Juscelino Kubitschek, was later elected President in 1955. Kubitschek worked with AIA to expand the ACAR model throughout Brazil by establishing the Brazilian Association of Rural Assistance and Credit (ABCAR – *Associação Brasileira de Crédito e Assistência Rural*). The establishment of a national network allowed for more centralized funding and direction. One of the principle functions of the aforementioned ETA was to scale up the experience of ACAR in Minas Gerais (see Mendonça, 2010). All existing regional and state extension agencies would then utilize the national administration to bolster their services. One example is the Northeastern Association of Credit and Rural Assistance (ANCAR – *Associação Nordestina de Crédito e Assistência Rural*). ANCAR used the ETA’s joint fund to import equipment and brought in nine extension advisors from the U.S. from 1955-1964 (Barber, 1965). Its director at the time was an economist named Dr. José Irineu Cabral, who would later become the first president of Embrapa (see Cabral, 2005).

Rural extension in Brazil was in many ways an institutional precursor to the redesign of agricultural research. The origins of Brazilian agricultural extension can be traced to the AIA pilot project in Minas Gerais that was based on the more traditional approach common in other Green Revolutions throughout the world by targeting family farmers. Rural extension was also a cornerstone of government and philanthropic collaboration in Mexico, the Andean region, and the Caribbean where agrarian reform had also taken place (Schultz, 1967). Agrarian reform was initially a priority of the Brazilian government in the late 1950s and early 1960s. However, after the military coup in 1964, the Brazilian state assured no structural changes to the agrarian sector and directed rural extension to work with large and medium producers under “conservative modernization”^5^. Therefore, while early approaches to rural extension in Brazil mirrored that of others in the region, the agrarian structure in Brazil remained largely unchanged.

In 1974 the Brazilian government created the Brazilian Company of Technical Assistance and Rural Extension, known by its Portuguese acronym Embrater. The newly-democratic government closed Embater in 1989 due to the combination of a fiscal crisis with the rise of social movements and public opposition to agricultural modernization (see Castro and Pereira, 2017). Agricultural extension in Brazil was once again relegated to state governments. Although rural extension in Brazil rarely receives credit for its role in the country’s agricultural development, the institution-building and relations behind its growth were fundamental to the vision of what would become Embrapa. Embrapa’s first two presidents and founders had both worked for ACAR and the state government of Minas Gerais. They received scholarships from the Rockefeller Foundation to train in the U.S. due to the relationships established while working with Nelson Rockefeller’s AIA in the state. These relationships with philanthropic organizations proved to be a crucial point of contact after returning to Brazil to help situate public research as the technological key for agricultural modernization.

### Public Agricultural Research

Extension services in Brazil grew out of the first public agricultural research institutions. Local extension services were established by the state governments of Bahia (1859), Pernambuco (1859), Sergipe (1860), and Rio de Janeiro (1860). However, these early research agencies were primarily tasked with on-farm extension and were lacking in any applied research. The first true research and development institute in Brazil was the Agronomical Institute of Campinas (IAC – *Instituto Agronômico de Campinas*), established in 1887 by Emperor Dom Pedro II. The IAC served as the de facto national research center for all things agricultural for several decades. Scientists at the center were responsible for the creation of some of the first new cultivars and conducted most of the first field trials. The first attempt to establish a national agricultural research network took place in 1933 but it only lasted a few years. Subsequent attempts in the 1930s and 1940s were also short-lived due to a lack of political stability and political will, and to the sector’s reliance on cheap labor. A conjuncture of international assistance and political stability in the following decades provided the underpinnings of a gradual institutionalization of a national agricultural research system for the country (see Rodrigues, 1987).

Nelson Rockefeller and his organizations played an important role early on in the establishment of agricultural research sites in Brazil. His scientists, who were contracted from U.S. universities, worked to transform seeds and soils in order to make the frontier amenable for industrial agriculture (Nehring, 2016; Márcio da Silva, 2013). The IBEC Research Institute (IRI) was created as a scientific research arm of his for-profit IBEC. IRI researchers looked for new areas of potential economic growth to serve the interests of IBEC’s corporate interests.^6^

It wasn’t until the military coup in 1964 that a marked change would occur for the government agencies focused on agricultural development. Military officials now in charge of governing the country determined that Brazil didn’t have an agrarian problem – in terms of land ownership and access – but rather a “rural problem.” What this meant was that rural areas were seen as underdeveloped only due to a lack of proper economic policy, infrastructure, and insufficient access to new technologies. Agricultural research, in particular, was prioritized by the military government over structural change in access to productive resources. Agrarian elites supported the military coup as they saw a future where the state would support the dispossession of lands to benefit large-scale, capital intensive agriculture (Bin, 2021).

The view of the Brazilian government was that a focus on staple grain production through large-scale agricultural expansion and modernization would, in the longer-term, generate the most state revenue and lower the price of domestic food prices for urban workers. At the same time, staple grain production at scale required significant capital and technology. A national rural credit scheme funded by USAID disproportionately benefitted larger producers for purchasing implements and inputs (see Helfand, 2001; Guedes, 1981; Spier, 2012). By 1975, only 5% of total credit contracts distributed 60.8% of total credit to large farms occupying almost 69% of total land (Gonçalves Neto, 1997: 175). The Brazilian government established price supports for staple crops such as soybeans, corn and wheat. But it was the technological inputs that were the most crucial if these staple crops from temperate climates and soils were to be grown at scale in the tropics.

The regime’s first agricultural development plan stated that the sector should “on the one side, provide real production surpluses for export in order to compensate for industrialized imports and, on the other, generate real monetary savings that enable capital formation” (Ministério da Agricultura, 1964). Agricultural industrialization was at the heart of this strategy and in the subsequent development plans of the military government. In order to do this, a High Level Commission on agricultural research was established to find ways to consolidate existing resources in the country and to focus the research agenda on commodity production for export.

Following the model of research centers organized around commodities and ecological regions, the military government established Embrapa on December 7^th^, 1972 (see Federal Government of Brazil, 1972). Immediately, technocrats in charge of Embrapa’s future development were convinced of the need to send researchers abroad for educational attainment (Duarte, 2016). One of the founders of Embrapa, Dr. Eliseu Alves, graduated from the agronomy department at UF-Viçosa and later earned a PhD in agricultural economics at Purdue University. Alves grew up on a farm in the state of Minas Gerais and his first job was as a rural extensionist in the budding state agency (ACAR) that was supported by Rockefeller’s AIA. Eliseu Alves, commented that at the first meeting of Embrapa its board of directors discussed the company’s initial strategy. Alves recalled that he said, “Brazil is the same size as the United States. Do you know what we need to do? The same thing as the U.S.!” (Alves, 2017). His mentioning of the similar sizes of the two countries is based on the seemingly comparable endowments of natural resources. However, the “thing” that was to be done by Alves and his contemporaries was to replicate the model of U.S. agricultural research and extension. According to their approach, any differences in land tenure structure or rural incomes would be corrected by technology generation and adoption in new production frontiers (see Alves et al., 2013).

Perhaps no one better exemplifies a Green Revolutionary than Alves. While at Purdue, he was supervised by Edward Schuh, an economist hired by the Ford Foundation to evaluate Brazilian agriculture in the 1950s. Schuh was convinced that the most important policy initiative for the Brazilian government was to invest in agricultural research and especially by training its staff abroad. The development of national scientific talent was important because Schuh did not think Brazil should import science and technology from “more advanced countries” because that “ignores the ecological specificity of the major fraction of agricultural research… [since] a more important problem in Brazil is the general lack of knowledge about tropical agriculture…” (Schuh, 1970: 420).

Schuh’s training left an important mark on Alves. Alves’ dissertation research at Purdue analyzed the optimal technological take-up of large-scale producers to modernize Brazilian agriculture (Alves, 1954). As a founding executive of Embrapa, he sought out international grants and loans with the World Bank, the Institute of International Education (IIE), and USAID to finance the postgraduate training of existing and incoming researchers. Academic department catalogs from U.S. universities were sent by the IIE to Embrapa headquarters. An internal Embrapa memo outlined the goal of sending 80 agronomists abroad per year for postgraduate training: “80% to the U.S. and 20% to “other countries – Latin America, Asia and Europe” (Embrapa, 1976). The World Bank research loans stipulated that the Bank would finance 100% of the costs associated with study in the U.S. and only 35% of costs for those who remained in Brazil (Embrapa/World Bank, 1981). From 1974 to 1980, Embrapa oversaw the postgraduate training of 1,349 researchers, with 176 PhDs obtained abroad and 65 within Brazil (Embrapa, 1981). The company established an English language training program in Brasília for its new recruits. Prior to departing the country, the recruits were required to attend a month of classes in the program (personal interview 10/12/2017).

Embrapa built on two decades of extensive international cooperation and diplomatic relations that was fundamental in extending its scientific network abroad. However, for the first time, Brazil had a unified *national* research network with political will and a clear research agenda for the short- and long-term. Embrapa constructed commodity research centers around the country and was established as a public company. The public company mandate allowed for relative autonomy in administering its internal affairs and in paying salaries that were competitive with the private sector. Most importantly, with the establishment of Embrapa, research became the government’s top priority for agricultural development, eclipsing both higher education and rural extension.

## Agricultural Research in Action: Inequality and Hybridity

One of the most common critiques of the Green Revolution concerns the effects it had on furthering income inequality and unequal access to productive resources. Economic inequalities were by and large increased during the adoption and nationalization of Green Revolution technology (c.f. Griffin, 1974: 225-229; Yapa, 1977; Freebairn, 1995). The same was also true in the Brazilian Green Revolution. Access to productive resources, especially land and farm implements, was exceptionally unequal in the country. The aggregate amount of cultivated land and the use of machinery increased significantly. In just under fifty years, the total planted land area more than doubled from 28.7 million hectares (ha) in 1960 to 59.8 million ha in 2006. The number of farm implements also increased tremendously: in 1960 there were only 61,000 in the country, which grew to over 1.2 million by 2017 (IBGE, 2020).

From 1950 to 1980, tied foreign assistance from USAID expanded the access to and use of fertilizers for large farms. Additional loans were provided for mineral surveys and the construction of mining infrastructure was geared towards fertilizer production (Nehring, 2016). Fertilizers were necessary for farmers who began planting the new crops that were promoted by commodity-specific credit and complimented by new seed varieties. Farms growing soybeans, in particular, increased their use of fertilizers from 38% to 89% of total farms from 1970-1980 (ibid: 31). The GINI index for the increasingly unequal concentration of land holdings also rose from .841 in 1960 to .859 in 1980 (Linhares and Teixeira da Silva, 1999: 170). By the consolidation of agricultural modernization in 1992, the proportion of total arable land by holding size fell to 15.4% for those of 100 ha or less and grew to 55.2% for farms of 1,000 ha or larger (INCRA, 1992).

Brazil’s distinct regions played a significant role in shaping the ways in which technologies were developed and deployed. The significant social, economic, and ecological differences between regions meant that some areas were targeted more than others for large-scale industrial production. The coast had long been more populated, with the South being a traditionally agricultural region while the Southeast and Northeast were largely extractive regions. Commodity cycles dominated agricultural production in the latter two regions. Coffee was predominately grown in the states of São Paulo, Rio de Janeiro, and Minas Gerais under a restrictive labor regime (Stolcke and Hall, 1983). During the 1950s, a drop in coffee prices combined with urbanization to result in relative labor scarcity for the first time since Portuguese colonization. Labor scarcity in coffee production was where Rockefeller’s IRI first found profitability through agricultural research. In 1952, on a 136,000-acre plantation near the town of Matão, in the state of São Paulo, IRI researchers started to experiment with weed control, plant breeding, soil correction, irrigation, chemical inputs, and mechanization. The experiment station of Matão was deep in the interior of the state and later became the center of IRI’s field plot experiments to transform the Cerrado into what would become one of the world’s most important soybean production zones (Márcio da Silva, 2018).

### Transnational Soybean Research

Recent literature on the ‘soy boom’ in South America has analyzed the causes of the boom and the consequences of the crop on national politics, the environment and rural livelihoods. Soybean production has expanded rapidly in countries such as Paraguay (Hetherington, 2020), Argentina (Leguizamon, 2020) and Bolivia (McKay, 2017) over the last several decades. Oliveira and Hecht (2016) include Brazil with those countries to constitute what they call “the United Soybean Republic or Soylandia” to explain the similarities in production models across the region. By pulling out the case of Brazil, this section intends to demonstrate the role of both public and private transnational research that underpinned the tropicalization of soybeans in the region and propelled Brazilian soy producers as some of the most competitive in the world. Indeed, while “Soylandia” may share similar historical connections and systems of production, the research behind soybeans in the tropics was unique to the Brazilian Green Revolution.

Soybeans were introduced to Brazil in the late 19^th^ century from the U.S. by Gustavo D’utra. He brought them to the low latitudes in the state of Bahia where they were unsuccessful due to light sensitivity. It wasn’t until 1908 that Japanese immigrants started producing soybeans with success in the south of the country where longer days allowed for the crop to sprout. The first documented introduction of cultivars for research happened when a researcher brought 48 varieties from the United States in 1926 (Bonato and Bonato, 1987). However, sanctioned public research on the crop didn’t begin until the 1930s and a government-approved cultivar was only launched in 1960, called “Pioneira” (Pioneer) (Feres and Gomes, 1981). This was soon to change with the institutional relationship between the U.S. and Brazil.

Both Rockefeller’s researchers at IRI and the Purdue-Viçosa agreement sought to facilitate soybean research in Brazil. According to one of the early U.S. plant breeders who worked in Brazil for Rockefeller’s IRI, soybeans were the focus of neither his research nor his interest. The way he put it, “one day I was sitting in my office [at IRI] and we had a visitor from Rio [de Janeiro], from AID (USAID), and the story was that Brazilian agriculture was a mess but there were no restrictions on helping Brazilians with soybeans” (personal interview 10/30/2019). According to USAID policy (M.O. 1016.2), U.S. funds were not allowed to fund technical cooperation projects that could compete with crops that had political and economic importance in the U.S. The crops banned for international technical support were rice, sugar, wheat, vegetable oils, citrus fruits, cotton, coffee, and tobacco (NRC, 1977: 95-96). There was no embargo for international research on soybeans. Both Brazilian and U.S. agronomists also saw other promising signs for soybean production in Brazil. They envisions the nitrogen fixation properties of soybeans as a promising crop to grow in the degraded soils of former coffee plantations due to (see Márcio da Silva, 2018). The Brazilian government also view soybeans as a crop with a favorable export market that was also amenable to large-scale production (Oliveira, 2016). Existing human resources in Brazilian public research were mobilized to start a research program on soybeans.

Dr. J. Ralph Shay, the chair of botany and plant pathology at Purdue University, went to Brazil as part of the Purdue-Viçosa partnership in 1963. He was a consultant in the development of academic programs at UF-Viçosa and worked with another professor at Purdue, Dr. G.O. Mott, to push soybean research in Brazil. Dr. Mott was later hired by IRI to be director of research and worked with the Brazilian government to establish the National Soybean Commission. Dr. Henry Shands, also from the Purdue-Viçosa partnership, was appointed as the coordinator of the program (Hymowitz et al., 1968). It wasn’t until 1965 that an agreement between USAID and Brazil allowed for the official exchange of genetic material.

Cultivars were imported into Brazil from North American research centers, mainly from the southern U.S., to create hybrids. The traditional soybean-producing region in Brazil was located in the south and the southeast. Researchers worked primarily at experimental field plots and farms throughout the states of São Paulo, Rio Grande do Sul, and Paraná. The first Brazilian soybean variety, Santa Rosa, was launched at the First National Soybean Festival in 1966 (Schurtleff and Aoyagi, 2009: 452). Santa Rosa was the result of cross breeding varieties from the U.S. such as Bragg, Davis, Hill, Hood, Hardee, Pickett, Bienville, and Bossier, which were some of the most productive in the U.S. at the time. These also led to the creation of additional Brazilian varieties such as IAS 1, IAS 4, IAS 5, and Paraná, among the first to have early success. The plant breeder and entrepreneur Francisco Teresawa also worked to develop a notable soybean variety called FT Cristalina that was specifically adapted to the acidic soils of the Cerrado He received seeds from a US farmer named Thomas Owens who was experimenting with soybeans in the region (Márcio da Silva, 2018).

State-funded research on soybeans from the Brazilian side was led by Dr. Romeu Kiihl. Dr. Kiihl was the son of a tailor in Piracicaba, São Paulo and had an interest in agronomy. The U.S. researchers stationed there were introduced to him and selected him to be trained. According to one of the U.S. soybean researchers stationed there, “we decided that they [the collaboration project] needed to train breeders because there really weren’t any [Brazilians]. They brought him [Kiihl] to Campinas [São Paulo] and taught him English… the idea was to train Brazilians to replace us” (personal interview 10/28/2019). Dr. Kiihl has now become known as the “father of soybeans.” He studied at Mississippi State University with Dr. Edgar Hardwig, who originally adapted soybeans to the Southern U.S. (Chaddad, 2016). The key contribution Kiihl made was to the adaptability of soybeans to tropical latitudes and soils. He moved to Embrapa’s soybean research center in the state of Paraná in 1978 and began to assemble a research team.

His team successfully created a variety in 1980 that was highly productive in tropical latitudes. This variety – called “Doko” – was based on six crosses, three of which included some of the original introductions from the southern U.S. (Crocomo and Spehar, 1981). The Doko variety was the only variety that provided a financial return in the Cerrado in its first year and was then widely adopted by farmers (Embrapa, 2016). The expansion of soybeans during the last 40 years in Brazil is one of the most notable markers of agricultural modernization and expansion in the country. Much of this expansion occurred in the Cerrado, a biome that represented less than 15 percent of national production prior to the late 1970s and that now produces over 60 percent of the country total (Dall’Agnol, 2016).

The case of soybeans demonstrates the quite literal hybridity between international and national actors in the production of new agricultural technologies. The introduction of new varieties was based on the alignment between national development goals and foreign capital in Brazil. Still, agricultural modernization in Brazil was due to more than just the national political will to invest in the “right” scientific research. It was also due to more than the imposition or *transfer* of Western scientific knowledge and technology into Brazil. National scientific research on soybeans in Brazil helped underpin the modernization of soy production in the country because it was based on adaptation of genetic material. This adaptation was made possible by the financial support of international agencies and the foreign training of national scientists, but it was enacted by the Brazilian government’s interest in building research institutions and the interest of large-scale farmers in expanding production on new lands.

## Conclusion

The triad of higher education, extension, and research became synonymous with agricultural development in the U.S. (Lourenço et al., 2020). It served as the model for the Green Revolution institutionally, scientifically, and culturally (Olsson, 2017). However, it wasn’t until the nationalization and consolidation of agricultural research in Brazil that genetic material was adapted to the ecological conditions in the country and industrialized at scale. Embrapa has stratgically linked the rapid modernization of Brazilian agriculture to what Lídia Cabral (2020) calls its “institutional heritage”. According to Cabral, Embrapa’s branding strategy of labeling itself as an engine of modernizing Brazilian agriculture continues to define the agency’s identity, which limits the viability of scientific alternatives (i.e. agroecology). Yet, behind this image as a national champion is a history of foreign interests and transnational research that helped modernize agriculture in the country.

Brazil’s Green Revolution is defined by the alignment of interests and actions between U.S. and national actors in modernizing Brazilian agriculture without restructuring the country’s agrarian structure. The military coup in 1964 consolidated agrarian elite interests around limiting (redistributive) agrarian reform and focusing on agricultural research to support large-scale industrial production. Ultimately, a focus on soybeans as a strategic crop helped to situate Brazil as one of the world’s largest soy producers and a key country for tropical agricultural research. Support from the Rockefeller’s private organizations and his role as a government official make this relationship unique in relation to other cases in the Green Revolution. The case of Brazil is also exceptional in demonstrating how a mutually reinforcing relationship to modernize agriculture overcame institutional shortcomings that have been identified elsewhere.

A.H. Moseman, a USAID administrator from 1965-67, foresaw these institutional shortcomings. According to Moseman (1966), USAID projects were “not always oriented to the problems of the country or to the development process itself. These institutions were not necessarily associated into *national* systems of research and development” (99, emphasis added). He also added that climates and ecologies were not considered by U.S. actors:

“misled by the success of the Marshall Plan… [is where] we found it very easy to transfer this particular [corn] hybrid into Western Europe. We failed to remember that the agriculture in northern Europe is similar to that of our Temperate Zone conditions, and that we can make a lateral latitudinal transfer” (Moseman, 1966: 99).

Exactly 40 years after Moseman’s statement at the 1966 Symposium on Research in Agriculture, Embrapa’s president, Silvio Crestana, made similar claims that Brazil had become the technological lynchpin connecting temperate and tropical agriculture. In his “borderless” world of free technological exchange, what Brazil had accomplished was now available to other tropical regions to industrialize agricultural commodity production. However, the transnational scientific relations behind the highly unequal process of the Brazilian Green Revolution is again left out in that claim. It was precisely this nationalization and consolidation of agricultural research that ensured a particularly “Brazilian” path of the Green Revolution.

## Figures and Tables

**Map 1:**
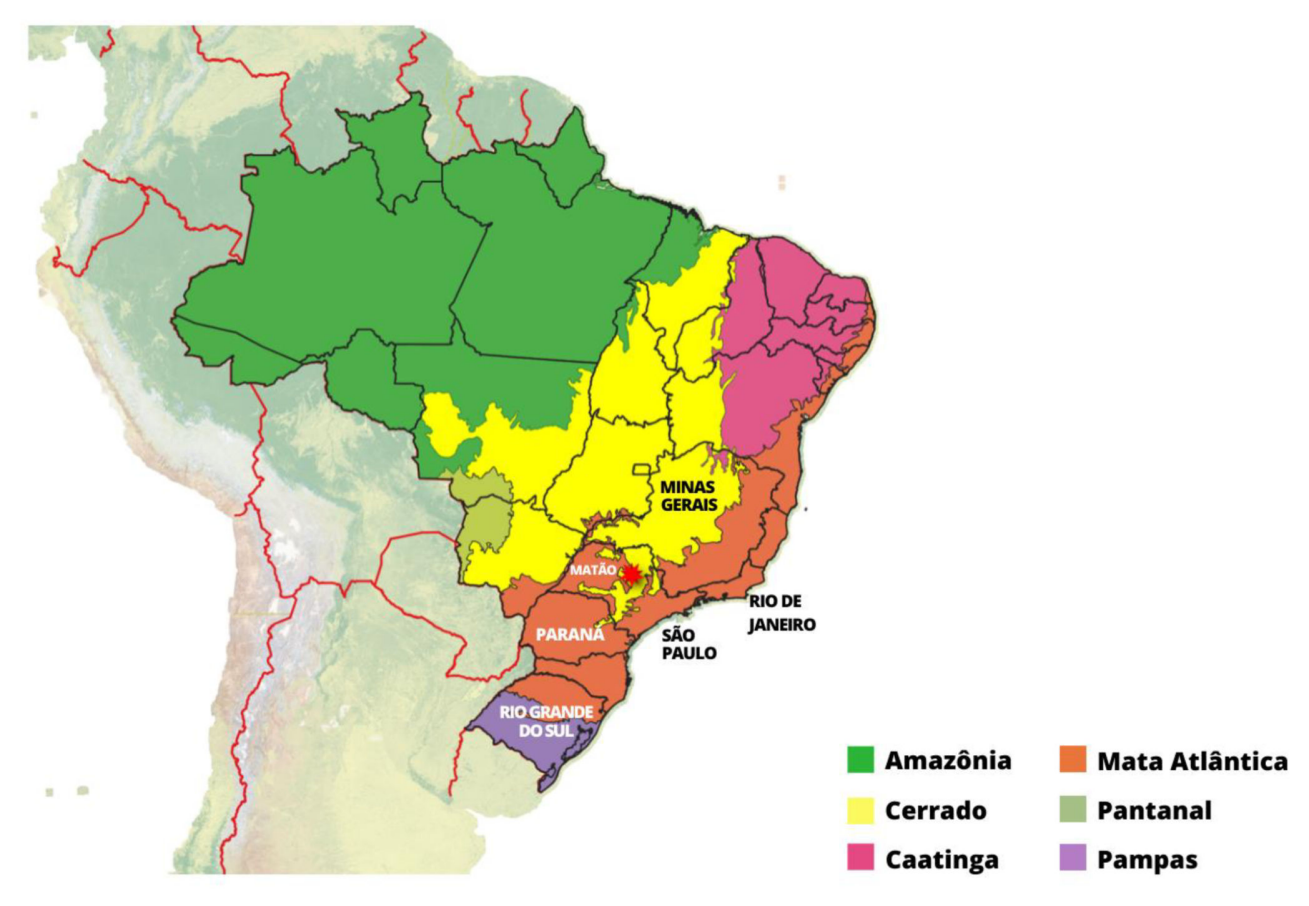
Brazilian Biomes and Research Sites Created by author using QGIS

## Footnotes

1 Silvio Crestana’s full speech and presentation can be found at: https://www.worldfoodprize.org/en/borlaug_dialogue/previous_years/2006_borlaug_dialogue/2006_transcripts_by_agenda/

2 Embrapa’s international laboratories began to expand into developing countries in the early 2000s, but they really exploded around 2010 with a focus on activities in Sub-Saharan Africa (Cabral et al., 2013).

3 While the Purdue-Viçosa partnership was the longest lasting, and arguably most influential, other partnerships expanded to include Ohio State-ESALQ, University of Arizona-Federal University of Ceará, and University of Wisconsin-Federal University of Rio Grande do Sul (see Sanders et al., 1989; and Sarruge Molina, 2016 on ESALQ).

4 The 4 H’s in North America are: Head, Heart, Hands and Health

5 In the case of rural extension, Reydon (1984) defines conservative modernization “in which participation of a national bourgeoisie, agrarian oligarchs and international capital, while excluding urban and rural working classes from participating” (4) (see also Pires and Ramos, 2009).

6 Rockefeller’s IBEC established five different companies in Brazil’s agricultural sector: a hybrid seed company, model farms for hog production, grain elevators, crop dusting by helicopter and airlines, and a mechanization company (importing implements) (see Broehl Jr., 1968: 48).
